# Clinical Utility of Circulating Tumor DNA in Advanced Rare Cancers

**DOI:** 10.3389/fonc.2021.732525

**Published:** 2021-11-24

**Authors:** Hitomi Sumiyoshi Okuma, Kan Yonemori, Yuki Kojima, Maki Tanioka, Kazuki Sudo, Emi Noguchi, Susumu Hijioka, Keiko Wakakuwa, Ken Kato, Akihiro Hirakawa, Aya Kuchiba, Takashi Kubo, Hitoshi Ichikawa, Akihiko Yoshida, Yasushi Yatabe, Kenichi Nakamura, Hiroyuki Mano, Noboru Yamamoto, Yasuhiro Fujiwara

**Affiliations:** ^1^ Department of Medical Oncology, National Cancer Center Hospital, Tokyo, Japan; ^2^ Clinical Research Support Office, National Cancer Center Hospital, Tokyo, Japan; ^3^ Department of Hepatobiliary and Pancreatic Oncology, National Cancer Center Hospital, Tokyo, Japan; ^4^ Department of Head and Neck Medical Oncology, National Cancer Center Hospital, Tokyo, Japan; ^5^ Division of Biostatistics and Data Science, Clinical Research Center, Tokyo Medical and Dental University, Tokyo, Japan; ^6^ Biostatistics Division, Center for Research Administration and Support, National Cancer Center, Tokyo, Japan; ^7^ Department of Clinical Genomics, National Cancer Center Research Institute, Tokyo, Japan; ^8^ Department of Diagnostic Pathology, National Cancer Center Hospital, Tokyo, Japan; ^9^ Division of Cellular Signaling, National Cancer Center Research Institute, Tokyo, Japan; ^10^ Department of Experimental Therapeutics, National Cancer Center Hospital, Tokyo, Japan; ^11^ Chief Executive, Pharmaceuticals and Medical Devices Agency, Tokyo, Japan

**Keywords:** rare cancer, CtDNA (circulating tumor DNA), precision medicine, soft tissue sarcoma, targeted therapy

## Abstract

**Purpose:**

Patients with advanced/relapsed rare cancers have few treatment options. Analysis of circulating tumor DNA in plasma may identify actionable genomic biomarkers using a non-invasive approach.

**Patients and Methods:**

Rare cancer patients underwent prospective plasma-based NGS testing. Tissue NGS to test concordance was also conducted. Plasma DNA alterations were assessed for incidence, functional impact, therapeutic implications, correlation to survival, and comparison with tissue NGS.

**Results:**

Ninety-eight patients were analyzed. Diseases included soft-tissue sarcoma, ovarian carcinoma, and others. Mean turn-around-time for results was 9.5 days. Seventy-six patients had detectable gene alterations in plasma, with a median of 2.8 alterations/patient. Sixty patients had a likely pathogenic alteration. Five received matched-therapy based on plasma NGS results. Two developed known resistance mutations while on targeted therapy. Patients with an alteration having VAF ≥5% had a significantly shorter survival compared to those of lower VAF. Tissue NGS results from eleven of 22 patients showed complete or partial concordance with plasma NGS.

**Conclusion:**

Plasma NGS testing is less invasive and capable of identifying alterations in advanced rare cancers in a clinically meaningful timeframe. It should be further studied as a prospective enrollment assay in interventional studies for patients with rare advanced stage cancers.

**Clinical Registration:**

[https://www.umin.ac.jp/ctr/index-j.htm], identifier UMIN000034394.

## Highlights

- Patients with advanced/relapsed rare cancers have limited understanding of genetic profiles that lead to treatment options, calling for an easier method to detect actionable alterations.
- Circulating tumor DNA (ctDNA) testing is less invasive and has proved to be clinically informative for many major cancers.
- Plasma NGS tests were performed on 98 rare cancer patients, alongside tissue NGS.
- Seventy-six patients had detectable gene alterations in plasma, with a median of 2.8 alterations per patient.
- Out of the 36 patients who had clinically informative alterations, five led to a matched therapy.
- Clinical outcomes in relation to the detected alterations were evaluated, including case reports of two rare cancer patients developing known resistance mutations while on targeted therapy.
- Our results demonstrate that it is feasible to detect actionable alterations in rare cancer patients in a clinically meaningful timeframe.

## Introduction

Multiplexed tissue-based genotyping has become standard of care in the diagnostic algorithm of patients with metastatic cancers. However repeated tissue biopsies are not feasible in many of these patients, especially for those with rare cancers, because of anatomical difficulties, existing comorbidities and/or clinical deterioration that necessitates rapid initiation of medical treatment. Patients with rare cancers have few treatment options due to a limited understanding of molecular characteristics and lack of clinical trials.

Plasma-based next generation sequencing (NGS) tests can detect cancer-derived DNA shed into the bloodstream (circulating-tumor DNA, ctDNA) and may be particularly useful in patients with rare cancers. Some ctDNA assays have demonstrated clinical validity and utility in certain types of advanced common cancers. For instance, the recent advent of ctDNA assays has drastically altered the diagnostic paradigm in non-small cell lung cancer (1, 2). However, there is insufficient evidence of clinical validity and utility for ctDNA assays in advanced rare cancers (3).

In the present study, we have carried out targeted plasma NGS in a prospective cohort of patients diagnosed with advanced rare cancers. Our primary objective was to demonstrate the clinical utility of plasma-based NGS.

## Methods

### Patients

Plasma NGS tests were performed on patients 16 years or older with advanced/metastatic rare solid tumors who matched the criteria for the MASTER KEY Project (4) and who provided written consent for plasma NGS testing between November 2018 to January 2019. The number of prior therapies was not restricted, allowing both pharmacotherapy-naïve and pharmacotherapy-treated patients to be included. Blood was collected prior to initial pharmacotherapy, at the time of disease progression, or while on treatment. Rare cancer was defined as cancer with an annual incidence *≤* 6/100,000 population in Japan. Clinical data were obtained from the patients’ electronic medical records. Among the patients who underwent plasma testing, those with available archival formalin-fixed paraffin-embedded (FFPE) tumor tissue specimens collected within six months of blood draw were also tested with tissue NGS.

The primary endpoint of the study was the alteration detection rate by plasma NGS testing. Secondary endpoints included the congruence of the reported gene alterations between plasma NGS and tissue NGS testing, application of relevant treatment according to the alterations, response to treatment, and survival.

This study was approved by the National Cancer Center Institutional Review Board and was conducted in full concordance with the principles of the Declaration of Helsinki.

### Plasma NGS Testing

NGS assay of plasma DNA was carried out using Guardant360^®^ (Guardant Health, Inc, Redwood City, CA). Cell-free DNA was isolated from plasma of patients’ blood samples. Guardant360^®^ is a targeted, hybrid-capture-based NGS panel test which, at the time of the study, detected point mutations in 73 genes, insertion-deletion mutations (indels) in 23 genes, amplifications for 18 genes, and fusions of six genes (**Supplementary Figure S1**). Detailed protocols for DNA isolation, sequencing and data analysis have been reported (5). The detected gene alterations were then classified according to their actionable levels. Actionability was predicted based on potential sensitivity/resistance to either an approved targeted agent or an experimental targeted agent currently in clinical trials. Evidence levels were added to each gene alteration according to Clinical Practice Guidance for Next Generation Sequencing in Cancer Diagnosis and Treatment (6) using cancer genome knowledge databases, such as CanDL (https://candl.osu.edu/browse), Cancer Genome Interpreter (https://www.cancergenomeinterpreter.org/biomarkers), CIViC (https://civic.genome.wustl.edu/home), and OncoKB (https://www.oncokb.org/). The following levels of evidence were assigned to each gene alteration: level 1A, a Pharmaceuticals and Medical Devices Agency (PMDA)-approved biomarker for the tumor type; 1B, a United States FDA-approved biomarker for the tumor type (not approved by the PMDA) or a biomarker verified by a prospective molecularly driven clinical trial; 2A, a biomarker identified by subgroup analysis in a prospective clinical trial; 2B, an approved biomarker for a different tumor type or a biomarker with evidence supporting its clinical utility; 3A, a biomarker with evidence of proof-of-concept in at least one case report; 3B, a biomarker with evidence obtained from *in vitro*/*in vivo* experiments; and 4, other gene mutations in cancer. In the present study, gene alterations with evidence levels 1A-3A were judged as actionable for drug selection.

### Tissue NGS Testing

The NCC Oncopanel test was used to assess the gene alterations in tumor tissue. This is a hybridization capture-based NGS assay designed to detect mutations of the entire coding region, amplifications, and homozygous deletions of 114 genes, along with fusion of 12 oncogenes (**Supplementary Figure S2**). For the analysis, FFPE tumor tissues with tumor cell content 10% or higher were used. Specialized pathologists estimated tumor cell content by counting the nuclei of tumor and non-tumor cells within each tissue. Detailed protocols for DNA isolation, sequencing and data analysis have been described elsewhere (7).

Concordance between plasma NGS and tissue NGS assays was assessed by comparing only the gene alterations that can be detected by both assays. Patients were classified into 4 categories; “concordant 1” = all reported alterations (from both assays) are consistent, “concordant 2” = all reported alterations (from both assays) are undetectable on either assay, “partially concordant” = at least one reported alteration (from both assays) is consistent, “discordant” = none of the reported alterations is consistent (excluding alterations undetectable on either assay).

### Clinical Outcome

Response to treatment was assessed according to version 1.1 of the Response Evaluation Criteria in Solid Tumor (RECIST) (8). Survival analyses were defined by the time interval from the date of blood sample extraction to the date of the relevant event. For Overall Survival (OS), qualifying events were death or loss of follow-up. For Progression Free Survival (PFS), the event date was the earliest of the date of discontinuation of therapy, the date of disease progression, or the last known date of clinical contact (if the patient were lost to follow-up). For those patients who did not die/progress during the study period, the outcome was considered right-censored. Survival curves were estimated with the Kaplan–Meier product-limit method and compared using the log-rank test. Hazard ratios (HRs) for OS were calculated using the Cox regression model. All hypothesis testing was carried out at a two-sided significance level of alpha = 0.05. Data were analyzed using JMP Pro version 13.0.0 (SAS Institute, Cary, NC, USA).

## Results

### Sample and Test Characteristics

We collected plasma from 98 patients among the 100 who provided consent from November 2018 to March 2019. Two patients had transferred hospitals before blood collection after providing consent. Median age of the patients who provided samples was 53 years (range 17-84), and 21 of these (21%) had received no prior systemic therapy at the time of blood sample collection. Baseline patient characteristics are shown in **Table 1**. Cancer types included in the study were soft tissue sarcoma (STS) (n=39, 40%), ovarian carcinoma (n=11, 11%), salivary gland carcinoma (n=9, 9%), neuroendocrine tumors (n=7, 7%), and 17 other types of rare cancers (**Supplementary Figure S3**). Subtypes of STSs included Ewing sarcoma (n=5), intimal sarcoma (n=4), synovial sarcoma (n=2), and alveolar soft part sarcoma (n=2).

**Table 1:** Baseline characteristics of all patients.

Characteristic	N (%)	Gene alteration positive (%)	Gene alteration negative (%)	0 < VAF < 5%	VAF ≥ 5%
Total	98	76 (78)	22 (22)	58	18
Gender					d
Male	40 (41)	31 (78)	9 (22)	22	9
Female	58 (59)	45 (78)	13 (22)	36	9
Median age (range)	53 years (17-84)				
<53	48 (49)	33 (69)	15 (31)	23	10
≥53	50 (51)	43 (86)	7 (14)	35	8
ECOG Performance status					
0	56 (57)	41 (73)	15 (27)	35	6
1	41 (42)	34 (83)	7 (17)	23	11
3	1 (1)	1 (100)	0	0	1
average turn-around-time (range)	9.5 days (5-20)				
Pharmacotherapy treatment status, Average (range)	1.6 (0-7)				
Treatment naïve	21 (21)	16 (76)	5 (24)	12	4
1	35 (36)	23 (66)	12 (34)	18	5
≥2	42 (43)	36 (86)	5 (14)	28	9
Tumor type					
Sarcoma	42 (43)	30 (71)	12 (29)	23	7
Non-sarcoma	56 (57)	46 (82)	10 (18)	35	11
Liver metastasis					
Yes	31 (32)	27 (87)	4 (13)	19	8
No	67 (68)	49 (73)	18 (27)	39	10
Lung metastasis					
Yes	40 (41)	31 (78)	9 (22)	23	8
No	58 (59)	45 (78)	13 (22)	35	10
Brain metastasis					
Yes	2 (2)	1 (50)	1 (50)	1	0
No	96 (98)	76 (79)	21 (21)	57	18
Bone metastasis					
Yes	25 (26)	18 (72)	7 (28)	11	7
No	73 (74)	58 (79)	15 (21)	47	11
Extra-regional lymph nodes					
Yes	23 (23)	18 (78)	5 (22)	11	7
No	75 (77)	58 (77)	17 (23)	47	11
Regional dissemination only					
Yes	18 (18)	15 (83)	3 (17)	12	3
No	80 (82)	61 (76)	19 (24)	46	15
Patients with tissue samples within 6 months of ctDNA test	37 (38)				
Tissue NGS tested	22 (22)	17 (77)	5 (23)	14	3

### Genomic Features

**Figure 1:**
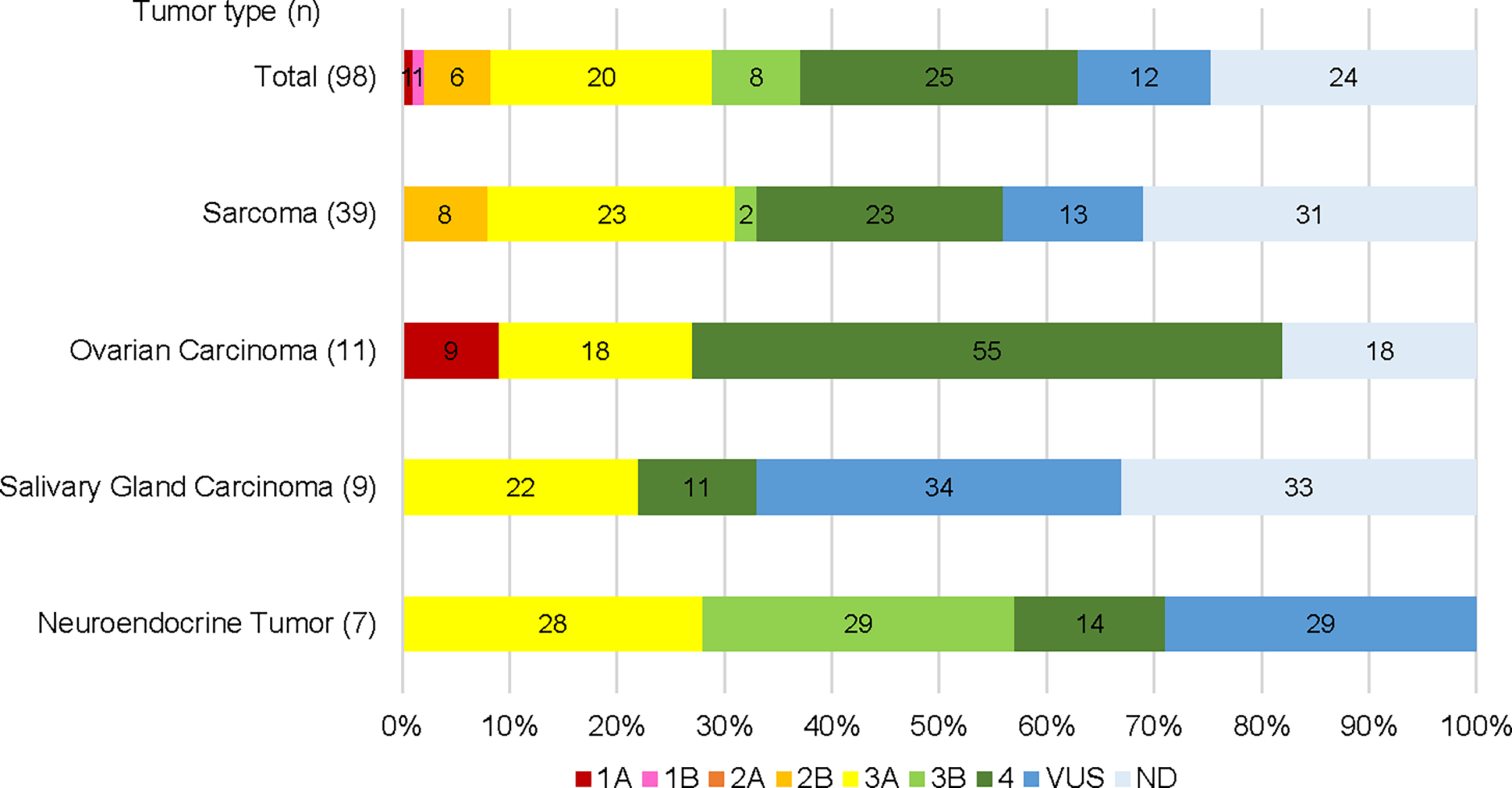
Gene alterations detected by plasma NGS testing categorized by potential clinical actionability evidence level. Total cases and top 4 cancer types are shown respectively.

Seventy-six patients (78%) had detectable gene alterations in plasma DNA (including variants of unknown significance and synonymous alterations). Sixty cases (61%) had at least one pathogenic alteration, 36 patients (48% of those with detectable gene alterations) had at least one actionable alteration, with evidence levels 1A-3A (**Figure 1**). Among tumor types with at least 7 representative cases in the sample, the actionable alteration rate was 31% (12/39) in STS, 27% (3/11) in ovarian carcinoma (including one patient with a level 1A gene alteration), 22% (2/9) in salivary gland carcinoma, and 28% (2/7) in neuroendocrine tumors (**Figure 1**).

**Figure 2:**
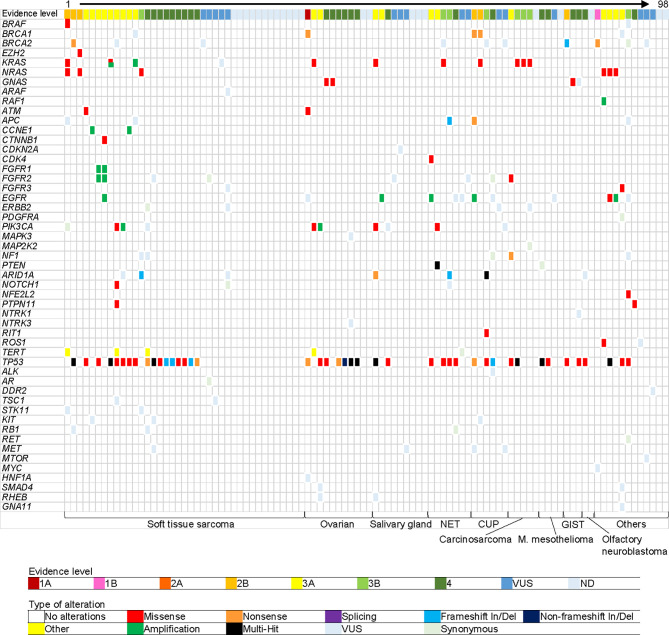
Gene alterations detected in plasma. Each column represents one patient. Each row represents one gene. Top row shows highest evidence level gene for each patient. Bottom row shows cancer type. Cancer types in “Others” are the following: pancreatic carcinoma (acinar cell), uterine carcinoma, thyroid carcinoma, melanoma, thymus carcinoma (squamous cell), adrenal cortical carcinoma, germ cell tumor, cholangiocarcinoma, gastrointestinal carcinoma (clear cell sarcoma-like), medulloblastoma, nephroblastoma, and NUT carcinoma, from left to right. VUS, variant of unknown significance; OS, overall survival; HR, hazard ratio; CI, confidence interval; and NE, not evaluable.

Thirty seven patients (38%) had co-occurring alterations (more than one pathogenic alteration were detected) (**Figure 2**). The most frequently affected gene was *TP53* (n = 43, 44%), of which 10 patients (12%, 10/43) showed multiple *TP53* mutations. Low allelic frequencies of some mutations in this multiple-hit population suggest a subclonal or hematopoietic source. The second most frequently affected gene was *KRAS* (n = 10, 10%), including one patient with a co-occurring *KRAS* G12V and *KRAS* amplification. Other commonly mutated genes included *NRAS* (n = 6, 6%), *EGFR* (n = 6, 6%), and *PIK3CA* (n = 6, 6%). The average turn-around-time, defined as the time from blood collection to receiving results, was 9.5 days (range 5-20).

### Clinical Outcome

Five of the 36 patients whose tumors had actionable alterations based solely on ctDNA findings received matched targeted therapies (**Supplementary Table S1**). Although none of these patients experienced an objective response, one with carcinoma of unknown primary and *EGFR* amplification (copy number 8.45) and treated with panitumumab showed tumor shrinkage in some of the metastatic lesions; however, treatment benefit for the remaining patients was limited. The main reason for not receiving therapies matched to their ctDNA results was the lack of access to the relevant medicine, either as standard of care or through a clinical trial (19/36). Others were either physically unfit for clinical trials due to rapid disease progression (6/36), had already been enrolled in a clinical trial according to biomarker results from tissue (3/36), failed to meet the biomarker inclusion criteria for a certain clinical trial when re-assessed by tumor tissue (1/36), changed hospitals (1/36), or were on a non-biomarker driven treatment at the time of follow-up (1/36).

**Figure 3:**
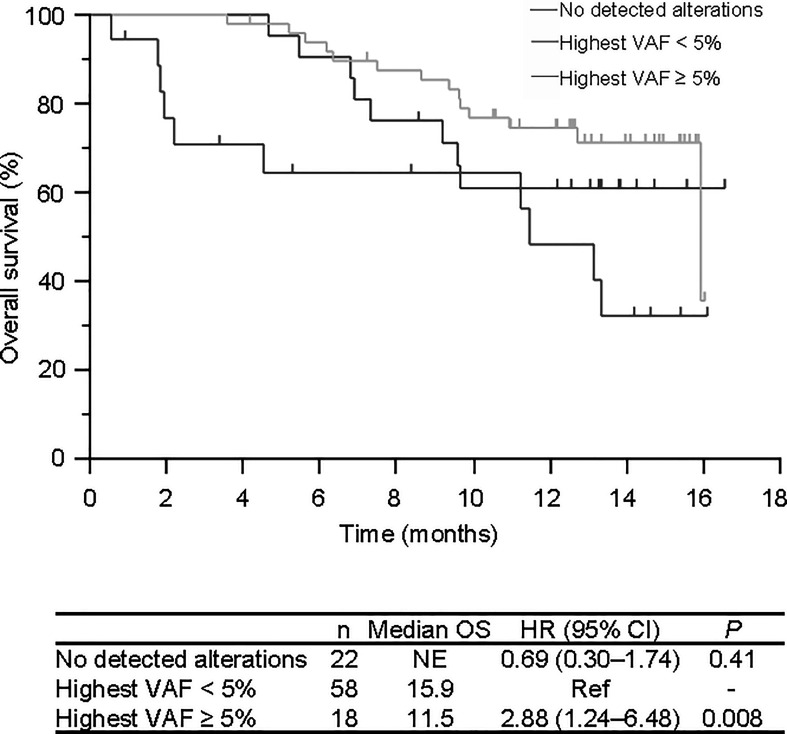
Overall survival of patients according to plasma NGS test results. No detected alterations in red, highest VAF of less than 5.0% in green, and highest VAF of 5.0% and over in blue. VAF, Variant allele frequency.

After a median follow-up of 11.8 months, the median OS was 15.9 months. The median OS was comparable between patients with at least one alteration and without any detectable alterations in plasma DNA (*P* =0.79; HR 1.2, 95% CI 0.510-2.81), but showed a significantly shorter survival in those with at least one alteration with a variant allele frequency (VAF) ≥ 5% (excluding confirmed germline mutations) (11.5 versus 15.9 months, *P* = 0.008; HR 2.88, 95% CI 1.24–6.48) (**Figure 3**). There was no significant difference between those with no alterations detected and those with a highest VAF < 5 (*P* = 0.41; HR 0.69, 95% CI 0.30–1.74) (**Figure 3**). We also performed a survival analysis between sarcomas and non-sarcomas but did not find a significant OS difference (data not shown).

**Figure 4:**
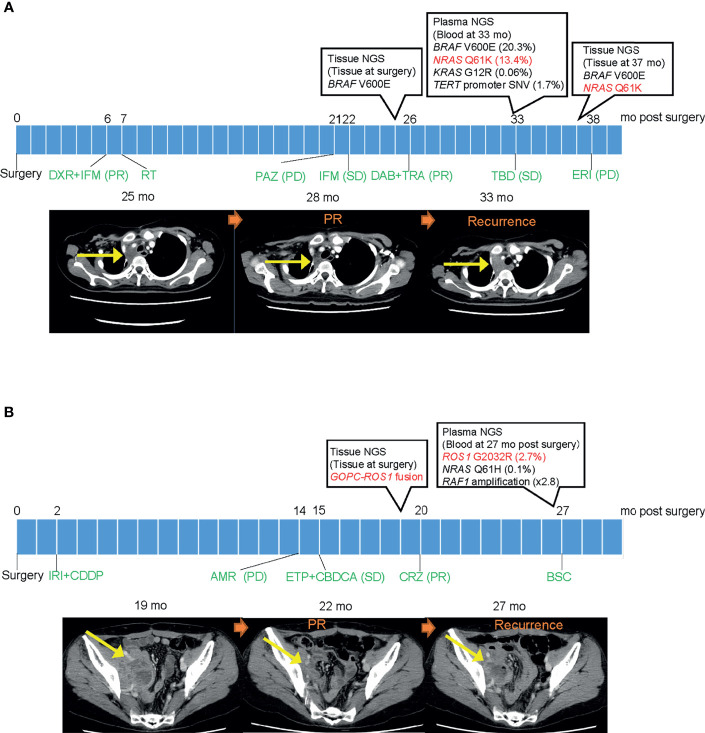
Clinical course of two patients with a detected mutation suggesting resistance. **(A)** Clinical course of patient with synovial sarcoma harboring *BRAF* V600E mutation. Left CT image shows a mass in the mediastinum before dabrafenib/trametinib treatment. Middle image shows reduction of tumor size after initiation of treatment. Right image shows recurrence after detecting *NRAS* Q61K resistant mutation in plasma DNA. **(B)** Clinical course of patient with adenocarcinoma mixed with neuroendocrine carcinoma of the uterus harboring *GOPC-ROS1* fusion. Left CT image shows a mass in the pelvic cavity before crizotinib treatment. Middle image shows reduction of tumor size after initiation of treatment. Right image shows recurrence after detecting *ROS1* G2032R resistant mutation in plasma DNA. PR, partial response; PD, progressive disease; SD, stable disease; mo, months; DXR, doxorubicin; IFM, ifosfamide; RT, radiotherapy; PAZ, pazopanib; DAB, dabrafenib; TRA, trametinib; TBD, trabectedin; ERI, eribulin; IRI, irinotecan; CPPD, cisplatin; AMR, amrubicin; ETP, etoposide; CBDCA, carboplatin; CRZ, crizotinib; and BSC, best supportive care.

Two patients had been receiving a targeted therapy at the time of plasma NGS testing, based on previous tissue NGS testing. One female patient in her 20s diagnosed with synovial sarcoma was found to have *BRAF* V600E mutation from a prior tissue NGS test and had received dabrafenib plus trametinib (9) (**Figure 4A**). Another female patient in her 40s diagnosed with adenocarcinoma mixed with neuroendocrine carcinoma of the uterus revealed *GOPC-ROS1* fusion from a prior tissue NGS test and had received crizotinib (**Figure 4B**). Tumors in both patients achieved a partial response. At the time of recurrence, both patients had plasma DNA testing that revealed acquired resistance mutations; *NRAS* Q61K and *ROS1* G2032R, respectively. The *ROS1* fusion in the latter patient was not detected by plasma NGS testing.

We also identified two other patients who were referred to the genetic counseling department because putative germline mutations from the plasma NGS tests were detected. *TP53* G245S (VAF 61%) and *BRCA2* Q3026* (VAF 30%) were detected from patients with leiomyosarcoma and pancreatic acinar cell carcinoma, respectively. With further confirmatory germline tests, both patients were diagnosed as having a hereditary cancer.

### Concordance With Tissue NGS

**Figure 5:**
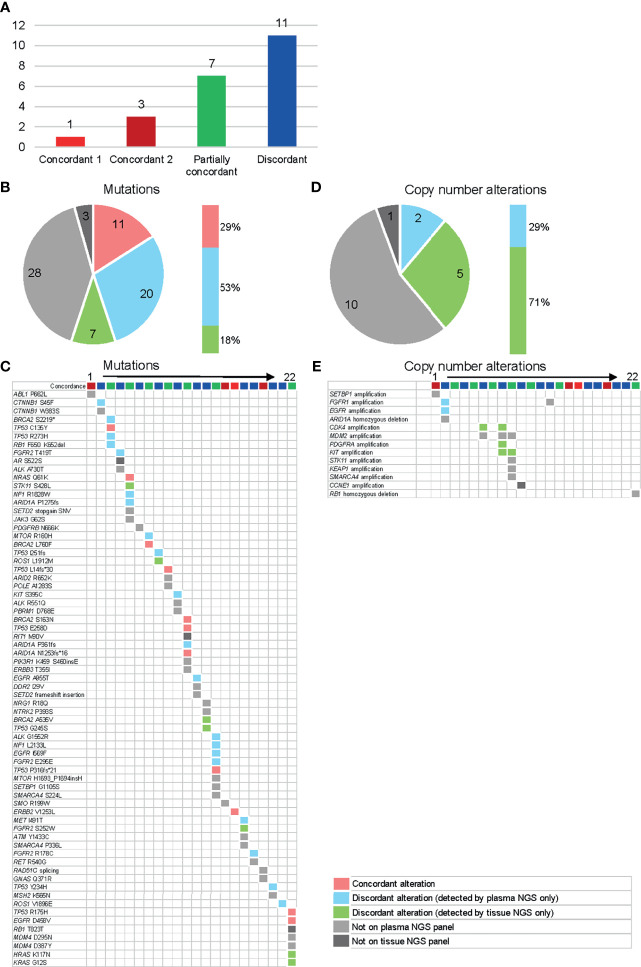
Concordance analysis of plasma and tissue NGS tests in 22 available patients. The color categories for Figures **(A–E)** are described in the lower right legend box. **(A)** Individual patient level concordance. Concordant 1 = all reported alterations (from both assays) are consistent, concordant 2 = all reported alterations (from both assays) are undetectable on either assay, partially concordant = at least one reported alteration (from both assays) is consistent, discordant = none of the reported alterations are consistent (excluding alterations undetectable on either assay). **(B)** Individual gene level concordance for the detected mutations. **(C)** Details of the detected mutations for each patient. **(D)** Gene level concordance for the detected copy number variants. **(E)** Details of the detected copy number variants for each patient.

Among the 35 patients who met the criteria of having a tissue biopsy within six months of blood collection, 22 patients were able to provide tissue samples within the required quantity and quality. Baseline characteristics of these patients are shown in **Supplementary Table S2**. In all, 11 patients (50%) showed some kind of concordance between the plasma NGS results and the tissue NGS results (concordance 1, concordance 2, or partially concordant) (**Figure 5A**). Only the alterations that can be detected in both tests were categorized as “comparable” and compared. Of the comparable mutations, 29% (11/38) were “concordant” and detected in both plasma and tissue DNA (**Figures 5B, C**). Fifty-three percent of all “comparable” mutations were reported only from plasma NGS. We further subclassified the patients into two categories and compared the detected genes (**Supplementary Figure S4**). In category 1, patients were classified into three groups according to time between tissue and blood collection: 1) < 30 days between tissue and blood collection date, 2) 30-120 days between tissue and blood collection date, 3) > 120 days between tissue and blood collection date. In category 2, patients were classified into two groups according to in-between pharmacotherapy status, 1) pharmacotherapy in between tissue and blood collection date, 2) no pharmacotherapy in between tissue and blood collection date. We did not observe a difference in concordance rate even when classified by time between tissue and blood collection date, nor did we observe a difference when classified according to in-between treatment. One main reason is the small number size in each group. The concordance of copy number alterations was very low. No consistent amplification was detected between plasma NGS and tissue NGS, with the majority (71%) being reported from tissue only (**Figures 5D, E**).

## Discussion

In the present study, we showed that NGS analysis of plasma DNA in rare cancers detects actionable variants that frequently co-occur with other potentially clinically relevant genomic alterations and has a potential to detect early resistance mutations and to predict patients’ prognosis. Research articles on ctDNA analysis on small groups of STS patients have been reported (10). With a larger cohort of patients, our study suggests the clinical utility of plasma NGS for rare cancer patients. Among the 76 patients with detectable gene alterations in plasma DNA, 36 (47%) were identified as having an actionable alteration. The detection rate of actionable alterations were comparable with prior reports using tissue NGS in rare cancer patients which range from 43-51% (11, 12). In our study, five patients (14% of patients with an actionable alteration) received genotype-matched therapies. This is an acceptable number when the target population is rare cancer patients who lack treatment choices of both standard of care and clinical trials. In the larger cohort of rare cancer patients from the MASTER KEY Project, 15% of patients received a “matched” therapy (4). Other studies have reported on a matched-therapy outcome of 8% (11, 13) using tissue NGS. The difference in the rates of patients receiving matched therapy might be a result of using different criteria to define “matched” therapy, the difference in the follow-up period, and/or the difference in available treatment options at the time of the study. The main reason for failing to receive a matched therapy to their plasma NGS results in the present study was the lack of treatment choices rather than clinical deterioration during the NGS test since the average turn-around-time was 9.5 days in our study. The failure rate to receive matched therapy due to clinical deterioration is generally higher using tissue-based NGS assays (14). Therefore, the present study suggested a need for a more efficient treatment development platform, such as the MASTER KEY Project (4), to bring more precision medicine options for rare cancers.

We also demonstrated a correlation between VAF levels in plasma DNA and OS in a cohort of rare cancer patients. This suggests that the ctDNA level in plasma DNA may reflect tumor burden, and patients whose tumors released higher amounts of ctDNA into plasma may have worse prognosis. Others have also reported similar findings, such as in a cohort of melanoma patients, the baseline plasma ctDNA level correlated with tumor volume, which suggested that ctDNA level may act as a surrogate biomarker for tumor burden and may be useful for following changes in tumor burden during treatment (15).

The two cases that detected resistance mutations taught us two important lessons. First, even in rare cancers where targeted therapy is not a common strategy yet, they show the same resistance mechanism as common cancers and therefore, biomarker testing at the time of disease progression may be able to guide treatment selection for the next line of therapy. Second, plasma NGS testing in a sequential manner would allow us to detect treatment failure before radiology tests in a rapid and non-invasive matter, giving us clues to the next treatment option. This is especially important for rare cancers where validated tumor markers have a limited role. The one case that detected *ROS1* G2032R was not able to receive further treatment due to limited choices of effective treatment at the time. However, now there are new promising selective tyrosine kinase inhibitors such as DS-6051b that overcome crizotinib resistant mutations (16).

The concordance between plasma NGS and tissue NGS has been a matter of discussion ever since plasma DNA became commonly used, and differences are seen between published papers. In common cancers such as NSCLC, publications have reported on an overall concordance of 81% when focused on one specific gene alteration such as EGFR, whereas the concordance rate was 53% when the concordance analysis included all alterations that were potentially detectable by both tests (i.e., included in both gene panels) as in our study (1, 17). Reasons for the discordance/partial concordance patients may be explained by intra-tumor heterogeneity, patients with local dissemination only with no hematogenous metastasis, or changes in gene profile due to chemotherapy between tumor specimen collection date and ctDNA blood collection date. Therefore, as for rare cancers, we believe that tissue biopsy remains essential for initial diagnosis, but ctDNA NGS is convenient in detecting alterations throughout the treatment course.

It must be noted that when dealing with plasma tests, incidental germline mutations must be considered. Close collaboration with the genetic counseling department is important. Through this research, we had two patients with possible germline mutations who were referred for genetic counseling. Both were diagnosed with germline mutations. According to a prior study, detection of putative germline mutations from plasma DNA was feasible across multiple genes and cancer types when selecting alterations of 40% or more VAF (18). In addition to these criteria, we were able to identify a germline *BRCA2* mutation with a VAF of 30%, suggesting that the cutoff of putative germline mutations may need further research.

Some limitations must be mentioned in the current study. First, the cohort included a wide range of cancer types and some cancer types were represented with only one patient. The cohort included both non-epithelial and epithelial tumors which present different clinical characteristics, which should be noted when interpreting the survival data between high VAF group and low VAF group. However, when the gene alteration detection rate and VAF levels were compared between sarcomas and non-sarcomas (**Table 1**), there was no significant difference, suggesting that plasma NGS is also a useful tool for sarcomas. Second, the number of patients who had both tissue NGS and plasma NGS tests were limited. This must be noted when interpreting the congruence data and should be verified with a larger group of patients.

In conclusion, potentially actionable alterations could be detected with plasma NGS, with relative ease considering its non-invasive nature and rapid TAT. Plasma NGS should be further studied as a prospective enrollment assay in interventional studies for patients with rare advanced stage cancers.

## Data Availability Statement

The data presented in the study are deposited in the National Library of Medicine NCBI Sequence Read Archive (SRA) submission: SUB10627377.

## Ethics Statement

The studies involving human participants were reviewed and approved by National Cancer Center Institutional Review Board (No. 2018-246). Written informed consent to participate in this study was provided by the participants’ legal guardian/next of kin. Written informed consent was obtained from the individual(s) for the publication of any potentially identifiable images or data included in this article.

## Author Contributions

HO: conception and design, writing, editing, revision, and approval of manuscript. KY: conception and design, writing, editing, revision, and approval of manuscript. YK: conception and design, writing, editing, revision, and approval of manuscript. MT: conception and design, writing, editing, revision, and approval of manuscript. KS: conception and design, writing, editing, revision, and approval of manuscript. EN: conception and design, writing, editing, revision, and approval of manuscript. SH: conception and design, writing, editing, revision, and approval of manuscript. KW: conception and design, writing, editing, revision, and approval of manuscript. KK: conception and design, writing, editing, revision, and approval of manuscript. AH: conception and design, writing, editing, revision, and approval of manuscript. AK: conception and design, writing, editing, revision, and approval of manuscript. TK: conception and design, writing, editing, revision, and approval of manuscript. HI: conception and design, writing, editing, revision, and approval of manuscript. AY: conception and design, writing, editing, revision, and approval of manuscript. YY: conception and design, writing, editing, revision, and approval of manuscript. KN: conception and design, writing, editing, revision, and approval of manuscript. HM: conception and design, writing, editing, revision, and approval of manuscript. NY: conception and design, writing, editing, revision, and approval of manuscript. YF: conception and design, writing, editing, revision, and approval of manuscript. All authors contributed to the article and approved the submitted version.

## Funding

Tissue NGS testing was supported in part by National Cancer Center Research and Development Fund (29-A-2).

## Conflict of Interest

KY reports personal fees from Eisai, AstraZeneca, Novartis, Ono Pharmaceutical, Pfizer, and Taiho Pharmaceutical, outside the submitted work. EN reports personal fees from Chugai Pharmaceutical, Eli Lilly, Pfizer, Sysmex, and AstraZeneca. AH reports grants and personal fees from Ono Pharmaceutical, personal fees from Astellas Pharma, Abbvie, Nippon Boehringer Ingelheim, Kissei Pharmaceutical, Pfizer, Nippon Shinyaku, Chugai Pharmaceutical, Taiho Pharmaceutical, Torii Pharmaceutical, Sumitomo Dainippon Pharma, Teijin Pharma, Fuji Pharma, Japan Tabacco, HEALIOS K.K, Life Science Institute, Inc., and Novartis, outside the submitted work. HI reports grants from Chugai Pharmaceutical, Eisai, Healios, and Ono Pharmaceutical, outside the submitted work. YY reports personal fees from MSD, Chugai Pharmaceutical, AstraZeneca, Novartis, Pfizer, Roche/Ventana, Agilent/Dako, and Thermo Fisher Science, outside the submitted work. NY reports research grants from Astellas, Chugai Pharmaceutical, Eisai, Taiho Pharmaceutical, Bristol Myers Squibb, Pfizer, Novartis, Eli Lilly, AbbVie, Daiichi-Sankyo, Bayer, Boehringer Ingelheim, Kyowa-Hakko Kirin, Takeda, Ono Pharmaceutical, Janssen Pharma, MSD, MERCK, GSK, and Sumitomo Dainippon, advisory roles from Eisai, Takeda, Otsuka, Boehringer Ingelheim, Cimic, and Chugai Pharmaceutical, and speaker rolls from Bristol Myers Squibb, Pfizer, AstraZeneca, Eli Lilly, Ono Pharmaceutical, Chugai Pharmaceutical, and Sysmex. HM reports a grant from Konica Minolta outside the submitted work. YF reports personal fees from AstraZeneca, Daiichi Sankyo, Chugai Pharmaceutical, Bristol Myers Squibb Japan, SRL, and Santen Pharmaceutical outside the submitted work.

The remaining authors declare that the research was conducted in the absence of any commercial or financial relationships that could be construed as a potential conflict of interest.

## Publisher’s Note

All claims expressed in this article are solely those of the authors and do not necessarily represent those of their affiliated organizations, or those of the publisher, the editors and the reviewers. Any product that may be evaluated in this article, or claim that may be made by its manufacturer, is not guaranteed or endorsed by the publisher.
