# Supplementary material for: Clinical Utility of Circulating Tumor DNA in Advanced Rare Cancers
Source: Front Oncol. 2021 Nov 24;11:732525. doi: 10.3389/fonc.2021.732525 (PMC8652216; doi:10.3389/fonc.2021.732525)

Supplementary Figures

- **Supplementary Figure S1:** Genes detected by Guardant360® V2.10 platform
- **Supplementary Figure S2:** Genes detected by NCC Oncopanel test
- **Supplementary Figure S3:** Number of enrolled patients by cancer type
- **Supplementary Figure S4:** Gene level concordance of detected mutations between plasma and tissue NGS tests when patients were sub classified according to two categories. Category 1; time between tissue and blood collection, category 2; in-between pharmacotherapy status. The color categories for alteration concordance are described in the lower right legend box. (A) Category 1-1) < 30 days between tissue and blood collection date (n=8), (B) Category 1-2) 30-120 days between tissue and blood collection date (n=9), and (C) Category 1-3) > 120 days between tissue and blood collection date (n=5). (D) Category 2-1) received pharmacotherapy in between tissue and blood collection date (n=16), and (E) Category 2-2) did not receive any pharmacotherapy in between tissue and blood collection date (n=6).

Supplementary Figure S1

Point Mutations – 73 Genes

AKT1	*ALK*	*APC*	*AR*	*ARAF*	*ARID1A*	*ATM*	*BRAF*	*BRCA1*	*BRCA2*
CCND1	*CCND2*	*CCNE1*	*CDH1*	*CDK4*	*CDK6*	*CDKN2A*	*CTNNB1*	*DDR2*	*EGFR*
ERBB2 (HER2)	*ESR1*	*EZH2*	*FBXW7*	*FGFR1*	*FGFR2*	*FGFR3*	*GATA3*	*GNA11*	*GNAQ*
GNAS	*HNF1A*	*HRAS*	*IDH1*	*IDH2*	*JAK2*	*JAK3*	*KIT*	*KRAS*	*MAP2K1 (MEK1)*
MAP2K2 (MEK2)	*MAPK1 (ERK2)*	*MAPK3 (ERK1)*	*MET*	*MLH1*	*MPL*	*MTOR*	*MYC*	*NF1*	*NFE2L2*
NOTCH1	*NPM1*	*NRAS*	*NTRK1*	*NTRK3*	*PDGFRA*	*PIK3CA*	*PTEN*	*PTPN11*	*RAF1*
RB1	*RET*	*RHEB*	*RHOA*	*RIT1*	*ROS1*	*SMAD4*	*SMO*	*STK11*	*TERT***
TP53	*TSC1*	*VHL*					** Includes *TERT* promoter region		

In-dels – 23 Genes

ATM	*APC*	*ARID1A*	*BRCA1*	*BRCA2*	*CDH1*	*CDKN2A*	*EGFR*	*ERBB2*	*GATA3*
KIT	*MET` ex14*	*MLH1*	*MTOR*	*NF1*	*PDGFRA*	*PTEN*	*RB1*	*SMAD4*	*STK11*
TP53	*TSC1*	*VHL*							

Amplifications – 18 Genes

AR	*BRAF*	*CCND1*	*CCND2*	*CCNE1*	*CDK4*	*CDK6*	*EGFR*	*ERBB2*
FGFR1	*FGFR2*	*KIT*	*KRAS*	*MET*	*MYC*	*PDGFRA*	*PIK3CA*	*RAF1*

Fusions – 6 Genes

ALK	*FGFR2*	*FGFR3*	*RET*	*ROS1*	*NTRK1*

MSI: High(or Not Detected)

Supplementary Figure S2

Mutations and copy number alterations for all exons					Fusions
ABL1	*CRKL*	*IDH2*	*NF1*	*RAC2*	*ALK*
ACTN4	*CREBBP*	*IGF1R*	*NFE2L2/Nrf2*	*RAD51C*	*AKT2*
AKT1	*CTNNB1*	*IGF2*	*NOTCH1*	*RAF1/CRAF*	*BRAF*
AKT2	*CUL3*	*IL7R*	*NOTCH2*	*RB1*	*ERBB4*
AKT3	*DDR2*	*JAK1*	*NOTCH3*	*RET*	*FGFR2*
ALK	*EGFR*	*JAK2*	*NRAS*	*RHOA*	*FGFR3*
APC	*ENO1*	*JAK3*	*NRG1*	*ROS1*	*NRG1*
ARAF	*EP300*	*KDM6A/UTX*	*NTRK1*	*SETBP1*	*NTRK1*
ARID1A	*ERBB2/HER2*	*KEAP1*	*NTRK2*	*SETD2*	*NTRK2*
ARID2	*ERBB3*	*KIT*	*NTRK3*	*SMAD4*	*PDGFRA*
ATM	*ERBB4*	*KRAS*	*NT5C2*	*SMARCA4/BRG1*	*RET*
AXIN1	*ESR1/ER*	*MAP2K1/MEK1*	*PALB2*	*SMARCB1*	*ROS1*
AXL	*EZH2*	*MAP2K2/MEK2*	*PBRM1*	*SMO*	
BAP1	*FBXW7*	*MAP2K4*	*PDGFRA*	*STAT3*	
BARD1	*FGFR1*	*MAP3K1*	*PDGFRB*	*STK11/LKB1*	
BCL2L11/BIM	*FGFR2*	*MAP3K4*	*PIK3CA*	*TP53*	
BRAF	*FGFR3*	*MDM2*	*PIK3R1*	*TSC1*	
BRCA1	*FGFR4*	*MDM4*	*PIK3R2*	*VHL*	
BRCA2	*FLT3*	*MET*	*POLD1*		
CCND1	*GNA11*	*MLH1*	*POLE*		
CD274/PD - L1	*GNAQ*	*MTOR*	*PRKCI*		
CDK4	*GNAS*	*MSH2*	*PTCH1*		
CDKN2A	*HRAS*	*MYC*	*PTEN*		
CHEK2	*IDH1*	*MYCN*	*RAC1*		

Supplementary Figure S3

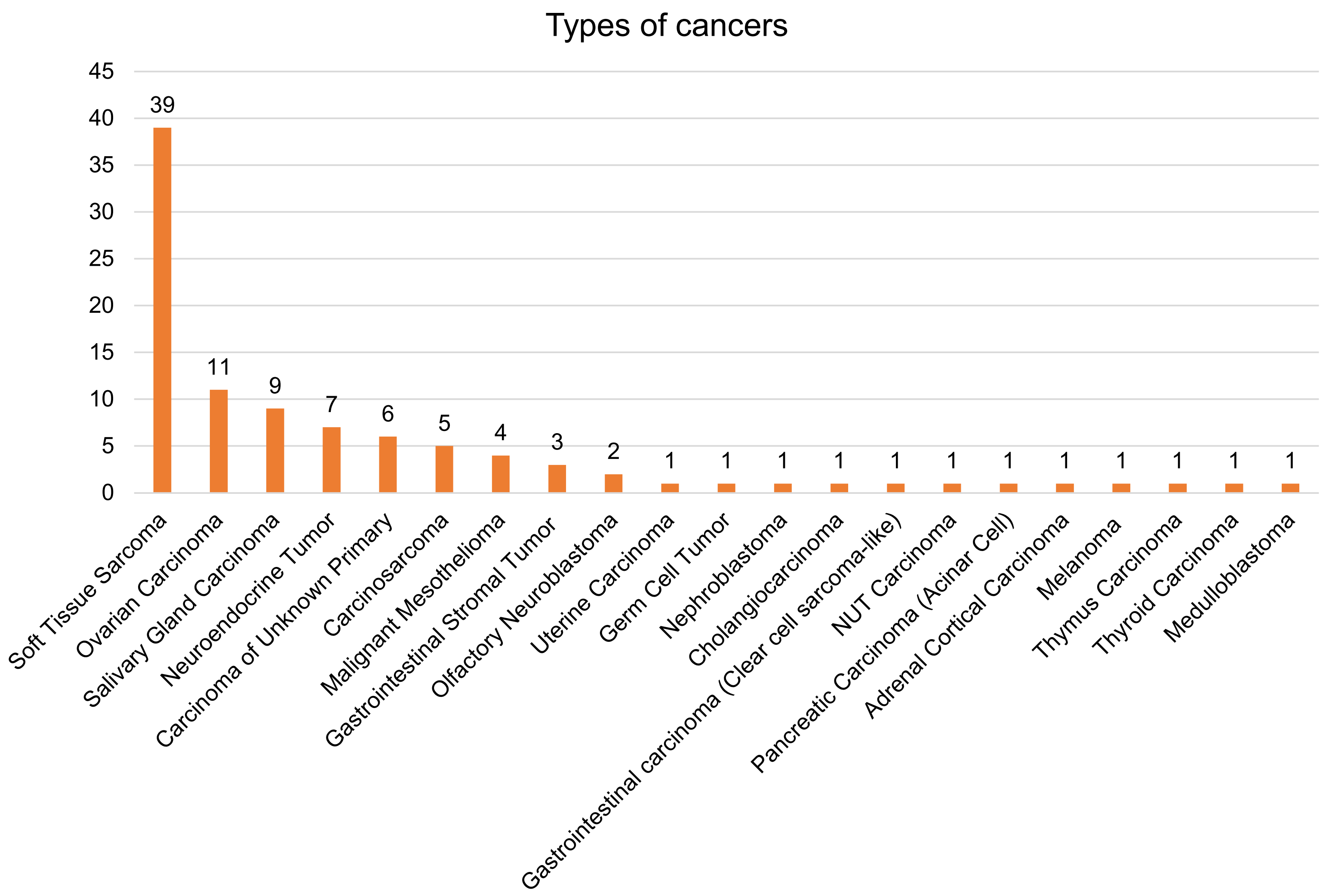

Supplementary Figure S4

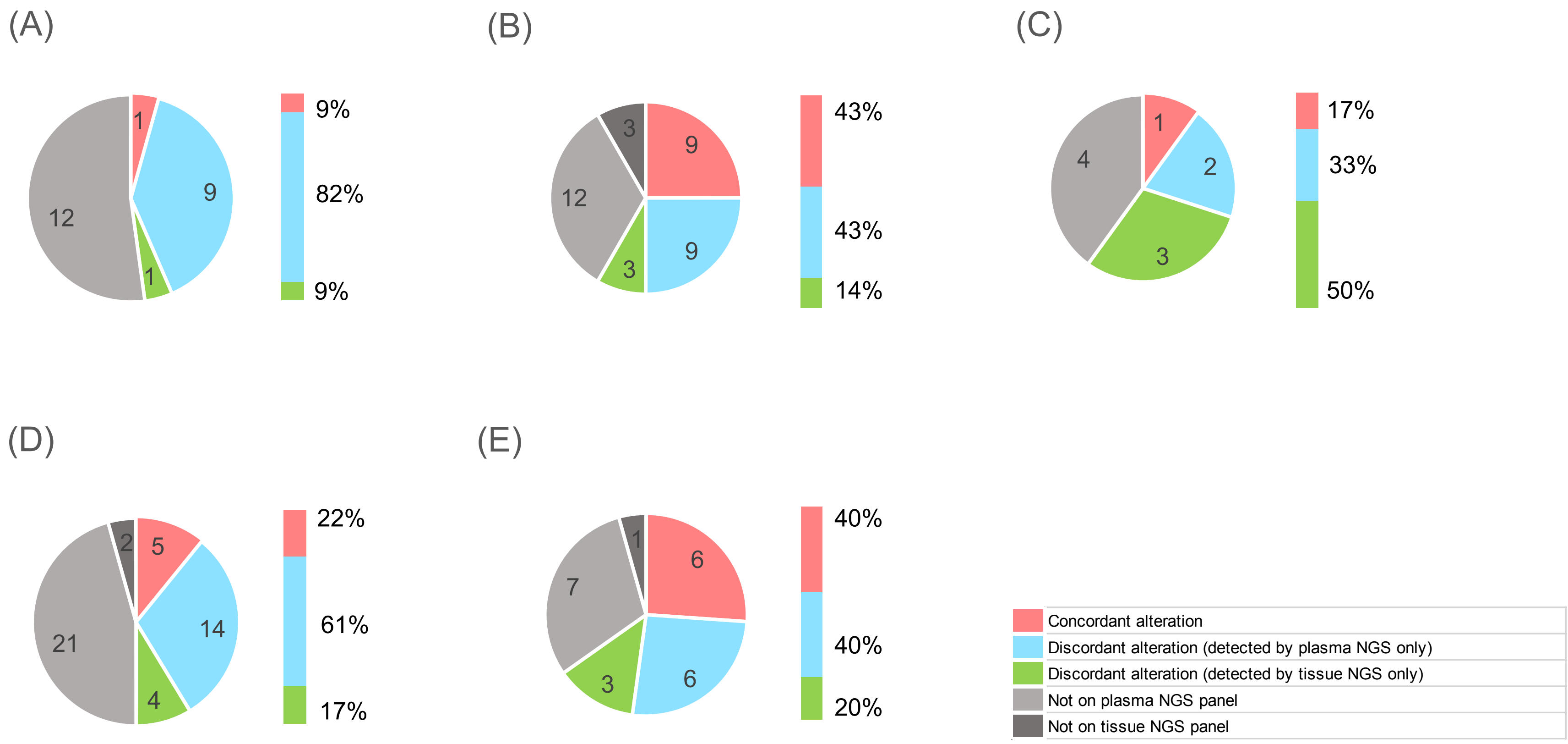

Supplement: Supplementary file 1 [file DataSheet_1.pdf]
