# Supplementary material for: Clinical Utility of Circulating Tumor DNA in Advanced Rare Cancers
Source: Front Oncol. 2021 Nov 24;11:732525. doi: 10.3389/fonc.2021.732525 (PMC8652216; doi:10.3389/fonc.2021.732525)
Supplement: Supplementary file 2 [file Table_1.docx]

Supplementary Table S1. Genotype-tailored therapies and their outcomes in patients with actionable alterations in ctDNA

| Patient No.*^1^ | Cancer type | Alteration | VAF or copy number | Co-mutations*^2^ | Treatment | Line of therapy | Treatment context | Best response | PFS (months) |
| --- | --- | --- | --- | --- | --- | --- | --- | --- | --- |
| 6 | Ewing sarcoma | FGFR1 (AMP) | x 2.61 | Yes | Pazopanib | Fourth | Standard care | PD | 5.1 |
| 52 | Adenoid cystic carcinoma | EGFR (AMP) | x 2.36 | No | Cetuximab | Fourth | Off-label | PD | 0.4 |
| 67 | CUP | EGFR (AMP) | x 8.45 | Yes | Panitumumab | Sixth | Off-label | PD (partial response in some lesions) | 4.3 |
| 68 | CUP | BRCA1 L63* | 46.51%*^3^ | Yes | PARP inhibitor | Second | Clinical trial | SD | 4.1 |
| 87 | Pancreatic acinar  cell carcinoma | BRCA2 Q3026* | 29.99%*^4^ | No | PARP inhibitor | Third | Clinical trial | PD | 2.8 |

*^1^Patient No. is in accordance with Figure 2 patient No.

*^2^co-occurring deleterious alterations (i.e. more than one pathogenic variant detected in ctDNA)

*^3^This patient had an existing germline *BRCA1* L63* mutation at the time of the plasma NGS test.

*^4^This patient was confirmed of germline *BRCA2* Q3026* mutation upon the plasma NGS test

CUP, carcinoma of unknown primary; AMP, amplification; VAF, variant allele frequency; PFS, progression-free survivalSupplementary Table S2. Characteristics of patients conducting tissue NGS

| Characteristic | N (%) |
| --- | --- |
| Total | 22 |
| Gender |  |
| Male | 9 (41) |
| Female | 13 (59) |
| Median age (range) | 53 years (23-72) |
| <53 | 10 (45) |
| ≥53 | 12 (55) |
| ECOG Performance status |  |
| 0 | 10 (45) |
| 1 | 11 (50) |
| 3 | 1 (5) |
| Pharmacotherapy treatment status, Average (range) | 0.64 (0-2) |
| Treatment naïve | 11 (50) |
| 1 | 8 (36) |
| 2 | 3 (14) |
| Liver metastasis |  |
| Yes | 5 (23) |
| No | 17 (77) |
| Lung metastasis |  |
| Yes | 6 (27) |
| No | 16 (73) |
| Brain metastasis |  |
| Yes | 1 (5) |
| No | 21 (95) |
| Bone metastasis |  |
| Yes | 6 (27) |
| No | 16 (73) |
| Extra-regional lymph nodes |  |
| Yes | 2 (91) |
| No | 20 (9) |
| Regional dissemination only |  |
| Yes | 6 (27) |
| No | 16 (73) |
| Time from tissue collection date to blood collection date (days) (median, range) | 58 (0-196) |
| <30 | 8 (36) |
| 31-120 | 9 (41) |
| 121- | 5 (23) |
| Treatment between tissue collection and blood collection |  |
| Yes | 6 (27) |
| No | 16 (73) |
